# The Correlation Between Attitudes Towards Health Technologies and Professional Self‐Efficacy in Intensive Care Nurses: A Cross‐Sectional Study

**DOI:** 10.1111/inr.70066

**Published:** 2025-07-08

**Authors:** Ayla Güllü, Keriman Aytekin Kanadli

**Affiliations:** ^1^ Faculty of Health Sciences Department of Nursing Obstetrics and Gynecology Nursing Department Hatay Mustafa Kemal University Antakya Hatay Turkey; ^2^ Hatay Mustafa Kemal University Hospital Education Coordination Antakya Hatay Turkey

**Keywords:** attitude, health technologies, intensive care, nurse, self‐efficacy

## Abstract

**Aim:**

The aim of this study is to determine the attitudes of intensive care nurses towards health technologies and their attitudes’ correlation with their professional self‐efficacy.

**Background:**

As the impact of digital technology in the field of health is rapidly increasing, intensive care units have become highly technologically advanced.

**Methods:**

A total of 90 nurses participated in this study. The data were gathered by using a Personal Information Form, the Health Personnel Health Technologies Assessment Attitude Scale (HPHTAAS) and the Nursing Profession Self‐Efficacy Scale (NPSES). The data were analysed using linear regression analysis, ANOVA and *t*‐test.

**Results:**

The findings of the study revealed that the HPHTAAS mean score of the intensive care nurses was 97.01 ± 9.23, and their NPSES mean score was 70.40 ± 6.29. The attitudes of the intensive care nurses towards health technologies and their professional self‐efficacy were significantly and positively correlated (*p* < 0.001). In addition, attitudes towards health technologies accounted for 24.9% of the variance of professional self‐efficacy.

**Conclusions:**

Intensive care nurses’ attitudes towards health technologies and professional self‐efficacy scores were above average.

**Implications for nursing and health policy:**

The evaluation of the effect of health technologies on nurses’ self‐efficacy can offer significant information to understand the process of their adaptation to technology and the effectiveness of health technologies. However, it is recommended to identify the deficiencies that hinder nurses from integrating new technologies into their professional activities and to organise necessary training programmes in respect thereof.

## 1 Introduction

Demographic evolutions and a growing number of comorbidities will increase the number of chronically ill patients who require intensive care treatment in the upcoming decades (Poncette et al. 2019). Moreover, healthcare organisations have encountered major challenges due to a shortage of medical staff, a growing workload, an increasing demand for care, and an increased financial burden. In this sense, rapid and sustainable implementation of advanced digital health technologies can help overcome these challenges, enhance the quality of care, and reduce healthcare costs (Brandsma et al. 2020; Poncette et al. 2019). Technological innovations have transformed the management and delivery of healthcare (Marques and Ferreira 2020). They allow healthcare professionals to establish early diagnosis in patients and make a timely intervention through remote monitoring, and also allow patients to have greater control and choice in their treatment (Sauchelli et al. 2023). Today, many health centres have adopted digital technology to enhance the quality of patient care and increase productivity and efficiency in the delivery of healthcare (Marques and Ferreira 2020). Novel technologies, known as artificial intelligence (AI), have been increasingly used in healthcare settings to process, interpret and refine the enormous amounts of data available and make transactions on them (Shinners et al. 2020).

Intensive care units (ICUs) have a long history of incorporating technology in patient care (Munro and Hope 2023). As one of the technologically advanced medical fields, ICUs are equipped with state‐of‐the‐art devices that can directly accomplish tasks delegated by caring clinicians (Poncette et al. 2019). Such devices include pumps that precisely deliver intravenous fluids and alert nurses against problems, as well as monitoring technologies that have been used for decades to recognise critical alterations in patients’ health conditions and guide daily intensive care treatment (Munro and Hope 2023; Poncette et al., 2019). Also, as information‐communication technologies and medical device technologies advance, new patient monitoring options have been introduced that can potentially improve patient safety, such as wireless monitoring sensors (ECG, pulse oximetry, and non‐invasive measurement of haemoglobin and hemodynamic parameters) and mobile communication devices (cell phones and tablets). However, AI‐based clinical decision support systems can assist physicians in identifying early symptoms of sepsis, respiratory failure, and bleeding by analysing multiple parameters (Poncette et al. 2019).

While new technologies promise to benefit the healthcare sector, the implementation of new technologies in the healthcare setting can be complex, and the acceptance of new technologies may differ between individuals and workplaces (Shinners et al. 2020; Stewart et al. 2024). Although advanced technology holds promise, negative attitudes towards the technology, unclear expectations and needs, and organisational problems may appear (Brandsma et al. 2020). AI producers aim to enable healthcare professionals to use digital health technologies safely in healthcare settings. According to Shinners et al. (2020), this requires determining the health professionals’ understanding and experience with new technologies, and the impact of the technology on their ability to deliver care (Shinners et al. 2020). Nurses, as frontline health professionals, may face new challenges and opportunities to adapt to productive technologies and use them to enhance the quality of care. Moreover, they bring insights into patient care and can meaningfully contribute to the development and adoption of relevant technology for health settings. Therefore, further research is required to explore how nurses perceive, adapt and utilise technologies in healthcare settings and how this affects their professional and personal well‐being (Singh and Kapoor 2024). To the best of our knowledge, there has been no study in the literature that examines the attitudes of intensive care nurses towards health technologies and their correlation with their professional self‐efficacy. From this point of view, the aim of this study is to examine the attitudes of nurses in ICUs, equipped with technology, towards health technologies and their attitudes’ correlation with their professional self‐efficacy, as health technologies have rapidly become widespread. For this purpose, an online survey was prepared and the hypotheses of the study were established.

### 1.1 Hypotheses

H1: The attitudes towards health technologies have a significant positive effect on professional self‐efficacy in intensive care nurses.

H2: The attitudes towards health technologies have a significant negative effect on professional self‐efficacy in intensive care nurses.

## 2 Methods

### 2.1 Type of the Study

This study was designed as descriptive, correlational and cross‐sectional.

### 2.2 Population and Sample

This study was conducted with nurses who were working in the ICUs of a hospital that renders tertiary health care services in a city located in the Southern Mediterranean region of Turkiye. Since intensive care nurses have to continuously improve their technological knowledge and skills in terms of patient safety and clinical effectiveness, this group was considered to be one of the most suitable groups to be included in this study. ICUs in the hospital are divided into different levels (levels 1–3) according to the health status of the patients. In Level 3 ICUs, healthcare professionals monitor and treat patients with serious, life‐threatening conditions. In Level 1 and 2 ICUs, doctors come to the unit to monitor the patients’ conditions when necessary. In Level 3 ICUs, a specialist doctor works 24 hours a day. The related hospital has a total of six intensive care units equipped with a total of 64 beds (number of beds in Level 2 ICUs: 14; number of beds in Level 3 ICUs: 50). The nurses in ICUs in this hospital work on three shifts (08–16, 16–08 and 08–08). The total number of nurses who work in these ICUs is 123. The sample size was calculated as 94 nurses using the calculation formula of a sample with a finite population, at a confidence interval of 95% and a margin of error of 0.05. It was determined that 20 nurses were on leave during the data collection stage. The data were collected by the researcher, who was working in the education unit of the related hospital and was also one of the authors of the present study. The relevant researcher visited the ICUs and informed the nurses about the study, sent the survey link online to the volunteers, and asked them to complete it at their convenience. The researcher stated that since the nurses worked in the same institution, the participants did not feel under pressure or obliged to participate. Inclusion criteria were determined as being 18 years of age or older, volunteering to participate in the study, and actively working in ICUs. Exclusion criteria were determined as not accepting to participate in the study and being on leave during the study time. The study was completed with 90 nurses who met the inclusion criteria. This sample size represented 73.17% of the population. Data were collected via an online survey (Google Forms) shared via WhatsApp between 1 and 16 July 2024.

### 2.3 Data Collection Tools

The data were collected using three data collection tools: a Personal Information Form, the Health Personnel Health Technologies Assessment Attitude Scale (HPHTAAS) and the Nursing Profession Self‐Efficacy Scale (NPSES).

#### 2.3.1 Personal Information Form

This form was prepared by the researchers to identify some descriptive characteristics of the participants and consisted of a total of eight questions (Kuscu et al. 2022).

#### 2.3.2 Health Personnel Health Technologies Assessment Attitude Scale (HPHTAAS)

The scale was developed by Kuscu et al. (2022) to determine the attitudes of healthcare professionals in Turkiye towards health technologies. The scale consists of 23 items and 3 subscales (scope, awareness and benefit). It has no reverse coding. ‘Scope subscale’ includes items 1 to 4, ‘awareness subscale’ includes items 5 to 11, and ‘benefit subscale’ includes items 12 to 23. Total score ranges between 23 and 115 points. Items in this 5‐point Likert scale are rated as ‘1, strongly disagree; 2, disagree; 3, undecided; 4, agree; 5, strongly agree’. The Cronbach's αvalue of the overall scale is 0.95, and the Cronbach's α values of the subscales range between 0.80 and 1.00 (Kuscu et al. 2022). In this study, Cronbach's α value of the HPHTAAS was found to be 0.929.

#### 2.3.3 The Nursing Profession Self‐Efficacy Scale (NPSES)

Caruso et al. (2016) developed the NPSES to assess nurses’ self‐efficacy to cope with difficulties in the workplace. This 5‐point Likert‐type scale consists of two subscales and 19 items (Caruso et al. 2016). All items are positive and are rated as ‘strongly agree 5, agree 4, undecided 3, disagree 2, and strongly disagree 1’. The Cronbach's α value of the scale is 0.83. Kacaroglu Vicdan and Tastekin (2019) adapted the scale into Turkish. The Turkish version of the scale includes two subscales and 16 items. Higher scores signify that the professional self‐efficacy improves. The Cronbach's α value of the Turkish version of the scale is 0.87 (Kacaroglu Vicdan and Tastekin 2019). In this study, Cronbach's α value of the NPSES was found to be 0.887.

### 2.4 Data Analysis

Analyses were done using the SPSS (IBM SPSS for Windows, ver. 24) statistical software. To analyse whether the data were normally distributed, skewness and kurtosis *Z* values were checked, and the values were found to be between −1.5 and +1.5 (see Table 2). According to Tabachnick and Fidell (2013), skewness and kurtosis values ranging between −1.5 and +1.5 indicate that the data did not deviate from normal distribution (Tabachnick and Fidell 2013). Since the data were normally distributed, a *t*‐test was used to analyse two variables, and an ANOVA test was used to analyse more than two variables. A post‐hoc LSD test was used to determine the difference between three or more groups. Simple linear regression analysis was run to determine the correlation between attitudes towards health technologies and professional self‐efficacy in intensive care nurses. The findings were evaluated at a confidence interval of 95% and *p* < 0.05.

### 2.5 Ethical Considerations

Ethics committee approval was obtained from Hatay Mustafa Kemal University Social and Human Sciences Scientific Research and Publication Ethics Committee (2 November 2022, Number: 11, Page: 3/5, Decision No: 16). Permission was obtained from the authors of the scales used in the study via e‐mail to use the scales in the study. Institutional permission was obtained from the related hospital. The consent of the participants was obtained by informing them about the study and asking for their consent before starting the data collection. The study adhered to the principles of the Declaration of Helsinki.

## 3 Results

The findings of the study indicated that 47.8% (*n* = 43) of the intensive care nurses were aged between 30 and 39 years, 78.9% (*n* = 71) were female, and 87.8% (*n* = 79) held a bachelor's degree. 64.4% of the participants were married, and 40% were working in the profession for 6–10 years (Table 1). When the institution‐related characteristics of the participants were analysed, it was observed that 35.6% of them were working in their current unit for 3–5 years, 73.3% were working on mixed shifts day and night, and 34.4% were working in the anaesthesia intensive care unit (Table 1).

**TABLE 1:** Descriptive and institution‐related characteristics of the participants.

Descriptive characteristics (*n* = 90)			Institution‐related characteristics (*n* = 90)		
Variables	*n*	%	Variables	*n*	%
**Age**			**Tenure in their current unit**		
18–29	42	46.7	0–2 years	5	5.6
30–39	43	47.8	3–5 years	32	35.6
40 and above	5	5.6	6–9 years	31	34.4
**Gender**			10 years and above	22	24.4
Female	71	78.9	**Manner of work**		
Male	19	21.1	Day shift	14	15.6
**Educational level**			Night shift	10	11.1
High school	4	4.4	Mixed shifts day and night	66	73.3
Associate degree	3	3.3	**Type of ICUs they worked in**		
Bachelor's degree	79	87.8	Internal medicine ICU	16	17.8
Master's degree	4	4.4	Surgical ICU	15	16.7
**Marital status**			Neurosurgery ICU	7	7.8
Single	32	35.6	Anaesthesia ICU	31	34.4
Married	58	64.4	Cardiovascular surgery ICU	10	11.1
**Duration of professional service (in years)**			Coronary ICU	11	12.2
0–5 years	28	31.1			
6–10 years	36	40.0			
11–15 years	20	22.2			
16 years and above	6	6.7			

Abbreviations: ICU, Intensive care unit; *n*, sample size.

Table 2 shows the descriptive statistics of the scales used in the study. The participants obtained a total score of 97.01 ± 9.23 from the HPHTAAS (confidence interval of 95%, 95.07–98.94); the mean score of HPHTAAS was calculated as 4.21 ± 0.40 (Likert‐type scale from 1 to 5). In this sense, it was found that the nurses’ attitude scores towards health technologies were above average. The total score for the ‘scope subscale’ of the HPHTAAS was calculated as 17.13 ± 1.83, with a mean score of 4.28 ± 0.45; the total score for the ‘awareness subscale’ was 31.32 ± 3.02, with a mean score of 4.47 ± 0.43; and the total score for the ‘benefit subscale’ was 48.55 ± 5.84, with a mean score of 4.04 ± 0.48. When the scores obtained from its subscales were examined, it was observed that the highest score was detected in the ‘awareness subscale’, followed by the ‘scope subscale’ and the ‘benefit subscale’, respectively.

**TABLE 2:** Descriptive statistics of the assessment tools.

Scales and subscales	Item no.	Mean *X* ± *SD*	Total *X* ± *SD*	95% CI	Min–Max	Skewness		Kurtosis	
HPHTAAS	23	4.21 ± 0.40	97.01 ± 9.23	95.07–98.94	74–115	0.470	0.254	−0.314	0.503
Scope subscale	4	4.28 ± 0.45	17.13 ± 1.83	16.74–17.51	12–20	−0.022	0.254	−0.244	0.503
Awareness subscale	7	4.47 ± 0.43	31.32 ± 3.02	30.68–31.95	25–35	−0.134	0.254	−1.323	0.503
Benefit subscale	12	4.04 ± 0.48	48.55 ± 5.84	47.33–49.78	33–60	0.472	0.254	0.218	0.503
NPSES	16	4.40 ± 0.39	70.40 ± 6.29	69.08–71.71	54–80	−0.173	0.254	−0.962	0.503

Abbreviations: HPHTAAS, Health Personnel Health Technologies Assessment Attitude Scale; NPSES, Nursing Profession Self‐Efficacy Scale, Likert‐style scale from 1 to 5; *SD*, Standard deviation; X, mean.

The total score obtained from the NPSES was 70.40 ± 6.29 (confidence interval of 95%: 69.08–71.71), and the mean score was 4.40 ± 0.39 (Likert‐type scale from 1 to 5). Accordingly, it was found that the professional self‐efficacy of the nurses was high (Table 2).

Table 3 shows the findings related to the participants’ HPHTAAS and NPSES scores according to their descriptive characteristics. The scores of the HPHTAAS, its subscales and the NPSES did not differ significantly (*p* > 0.05) according to the variables of age, gender, educational level, total years of professional service, tenure in the current unit, and the type of their ICU. The scores of the ‘awareness subscale’ of the HPHTAAS significantly differed in terms of the manner of work (shift). The score of those who worked on mixed shifts (day and night) on the ‘awareness subscale’ of HPHTAAS (31.80 ± 3.06) was higher than the score of those who worked on permanent night shifts (29.50 ± 2.36) (*p* = 0.033, *F* = 3.557). Furthermore, the manner of work did not significantly affect their NPSES scores (*p* > 0.05) (Table 3).

**TABLE 3:** Findings related to the HPHTAAS and NPSES scores of the participants.

Variables (*N* = 90)	HPHTAAS total score *X* ± *SD*	Scope subscale *X* ± *SD*	Awareness subscale *X* ± *SD*	Benefit subscale *X* ± *SD*	NPSES total score *X* ± *SD*
**Age, years**					
18–29	97.54 ± 9.23	17.35 ± 1.84	31.52 ± 2.89	48.66 ± 5.90	71.73 ± 6.08
30–39	96.83 ± 9.65	16.95 ± 1.87	31.32 ± 3.24	48.55 ± 6.07	68.97 ± 6.46
40 and above	94.00 ± 5.52	16.80 ± 1.30	29.60 ± 1.67	47.60 ± 3.91	71.40 ± 4.56
*p(F)* value	*0.713 (0.339)*	*0.552 (0.599)*	*0.409 (0.902)*	*0.930 (0.073)*	*0.121 (2.166)*
**Gender**					
Female	96.98 ± 9.18	17.15 ± 1.84	31.32 ± 3.05	48.50 ± 5.81	71.05 ± 6.20
Male	97.10 ± 9.66	17.05 ± 1.84	31.31 ± 3.00	48.73 ± 6.14	67.94 ± 6.18
*p(t)* value	*0.960 (0.050)*	*0.830 (0.215)*	*0.992 (0.010)*	*0.880 (0.151)*	*0.055 (1.941)*
**Educational level**					
High school‐ Associate degree	93.28 ± 6.21	16.71 ± 0.75	30.14 ± 2.54	46.42 ± 4.96	71.85 ± 4.74
Bachelor's degree and higher	97.32 ± 9.40	17.16 ± 1.89	31.42 ± 3.05	48.73 ± 5.90	70.27 ± 6.41
*p(t)* value	*0.269 (1.113)*	*0.220 (1.286)*	*0.285 (1.076)*	*0.319 (1.002)*	*0.527 (0.636)*
**Marital status**					
Single	97.81 ± 9.37	17.56 ± 1.66	31.50 ± 3.10	48.75 ± 5.78	70.71 ± 6.35
Married	96.56 ± 9.21	16.89 ± 1.88	31.22 ± 3.00	48.44 ± 5.93	70.22 ± 6.31
*p(t)* value	*0.544 (0.609)*	*0.099 (1.668)*	*0.681 (0.412)*	*0.816 (0.233)*	*0.723 (0.355)*
**Duration of professional service (in years)**					
0–5	99.39 ± 10.18	17.75 ± 1.73	31.89 ± 3.03	49.75 ± 6.55	71.60 ± 6.76
6–10	95.75 ± 8.79	16.94 ± 1.67	31.30 ± 3.05	47.50 ± 5.76	70.50 ± 6.57
11–15	96.25 ± 9.19	16.60 ± 2.18	30.85 ± 3.16	48.80 ± 5.38	68.15 ± 5.33
16 and above	96.00 ± 6.95	17.16 ± 1.47	30.33 ± 2.33	48.50 ± 4.13	71.66 ± 4.13
*p(F)* value	*0.439 (0.911)*	*0.153 (1.800)*	*0.553 (0.702)*	*0.505 (0.786)*	*0.283 (1.291)*
**Tenure in their current unit, years**					
0–5	98.72 ± 9.67	17.59 ± 1.72	31.70 ± 2.96	49.43 ± 6.17	71.27 ± 6.37
6–9	96.74 ± 9.93	16.96 ± 1.88	31.51 ± 3.37	48.25 ± 6.28	71.06 ± 6.75
10 and above	94.50 ± 6.93	16.59 ± 1.81	30.40 ± 2.50	47.50 ± 4.52	68.00 ± 5.00
*p(F)* value	*0.233 (1.483)*	*0.103 (2.334)*	*0.259 (1.371)*	*0.448 (0.811)*	*0.119 (2.181)*
**Manner of work**					
Day shift (1)	93.92 ± 5.73	17.00 ± 1.41	30.35 ± 2.64	46.57 ± 3.69	69.42 ± 4.50
Night shift (2)	93.80 ± 8.70	17.00 ± 1.41	29.50 ± 2.36	47.30 ± 5.67	66.90 ± 7.23
Mixed shift (3) 3>2	98.15 ± 9.73	17.18 ± 1.97	31.80 ± 3.06	49.16 ± 6.17	71.13 ± 6.35
*p(F)* value	*0.152 (1.927)*	*0.919 (0.085)*	***0.033*** ^*^ ***(3.557)***	*0.250 (1.409)*	*0.114 (2.223)*
**Type of ICUs they worked in**					
Internal medicine ICU	96.18 ± 9.73	16.68 ± 1.95	30.68 ± 3.57	48.81 ± 6.54	70.62 ± 6.90
Surgical ICU	98.33 ± 10.47	17.86 ± 1.76	31.73 ± 3.05	48.73 ± 6.69	70.53 ± 6.64
Neurosurgery ICU	99.85 ± 10.05	16.71 ± 2.05	32.14 ± 3.13	51.00 ± 5.35	69.85 ± 4.67
Anaesthesia ICU	96.80 ± 9.30	17.25 ± 1.76	31.25 ± 2.90	48.29 ± 5.71	70.58 ± 6.60
Cardiovascular surgery ICU	92.00 ± 5.24	16.40 ± 1.89	30.60 ± 2.71	45.00 ± 3.71	67.60 ± 6.31
Coronary ICU	99.72 ± 8.69	17.36 ± 1.62	32.00 ± 2.96	50.36 ± 5.40	72.27 ± 5.23
*p(F)* value	*0.422 (1.002)*	*0.347 (1.137)*	*0.766 (0.513)*	*0.302 (1.232)*	*0.702 (0.597)*

* *p* < 0.05.

Abbreviations: HPHTAAS, Health Personnel Health Technologies Assessment Attitude Scale; ICU, Intensive Care Unit; NPSES, Nursing Profession Self‐Efficacy Scale; *SD*, Standard deviation; X, mean.

According to the results of the regression analysis, the variables of HPHTAAS and NPSES were moderately and significantly correlated with each other (*R*: 0.499, *R* ^2^: 0.249, *p* < 0.001). HPHTAAS accounted for 24.9% of the total variance. In other words, the independent variable (HPHTAAS) included in the model accounted for variation in the dependent variable (NPSES) by 24.9%. According to the standardised regression coefficients (β), a one‐unit increment in attitude towards health technologies led to an increase of 49.9% in professional self‐efficacy. This result revealed the importance of encouraging positive attitudes towards health technologies to improve nurses’ professional self‐efficacy. When the *t*‐test results regarding the significance of the regression coefficients were analysed, it was found that HPHTAAS had a significant positive effect on NPSES (Table 4). Accordingly, hypothesis H1 (The attitudes towards health technologies have a significant positive effect on professional self‐efficacy in intensive care nurses) was accepted.

**TABLE 4:** Results of regression analysis for attitude towards health technologies and professional self‐efficacy.

Variables	*B*	St. error	*β*	*t*	*p*
**(Constant)**	37.395	6.136		6.094	0.000^*^
**HPHTAAS**	0.340	0.063	0.499	5.403	0.000^*^
**Dependent variable: NPSES**					
*R*: 0.499	*R* ^2^: 0.249	*F*: 29.189	*p*: 0.000^*^	Durbin Watson: 1.878	

* *p* < 0.001.

Abbreviations: HPHTAAS, Health Personnel Health Technologies Assessment Attitude Scale, NPSES: Nursing Profession Self‐Efficacy Scale.

## 4 Discussion

Intensive care nurses play a critical role in ensuring patients receive care continuously. They are required to monitor vital signs, administer complex treatments and respond quickly to dynamic patient conditions. Self‐efficacy refers to the ability of individuals to accomplish a specific task or a goal and is crucial in how individuals approach and cope with challenging circumstances (Lin et al. 2024). Technological advances have led to a significant change in the organisation and structure of the nursing profession and have been transforming the nursing industry with new delivery methods in modern health care (Tarsuslu et al. 2024). Therefore, it is important to determine nurses’ attitudes towards technological innovations. This study was conducted to determine the attitudes of intensive care nurses towards digital health technologies and their correlation with their professional self‐efficacy. The HPHTAAS and NPSES total scores of the intensive care nurses were found to be 97.01 ± 9.23 and 70.40 ± 6.29, respectively, in the study. Based on this result, it can be asserted that the intensive care nurses in the sample group exhibited positive attitudes towards technologies in the health field and had high professional self‐efficacy. A study conducted with nurses who worked in hospitals in three regions of Turkiye reported that 82.7% of the nurses held positive attitudes towards AI (Tarsuslu et al. 2024). Another study indicated that healthcare professionals were generally well aware and had an optimistic attitude towards AI (Surbaya et al. 2024). On the other hand, a previous study reported that healthcare providers considered the digital health monitoring experience as satisfactory (P et al. 2024). This study is compatible with the literature. On the other hand, other studies revealed that existing technologies failed to meet the expectations of healthcare professionals and most of nurses lacked a solid understanding of AI, wanted to learn more about new technologies, and they needed more time to learn (Golz et al. 2022; Poncette et al. 2020; Sommer et al. 2024). Transparency, understandability, technical soundness, interoperability, usability and staff training are essential to promoting the use of AI (Poncette et al. 2020; Shevtsova et al. 2024). As primary users of medical AI technology, nurses need to follow developments in the field (Shevtsova et al. 2024). Nurses need digital competencies and training to use AI safely and effectively, to engage in its implementation, planning and evaluation and to integrate new technology into their professional activities (Jobst et al. 2022; Reading Turchioe et al. 2024). However, it is stated in the literature that while advanced technologies allow nurses to focus their time and energy directly on patient care and reduce their workload, there are some potential disadvantages of technology, such as the inability to show human emotions and cultural sensitivity (Liang et al. 2019). Nurses need to protect patient autonomy and provide respectful patient care when using health technologies. This issue should also be emphasised in the training provided.

In this study, it was observed that the scores obtained from the HPHTAAS subscales were above average, and the highest score was detected in the ‘awareness subscale’. The awareness subscale includes statements indicating that health technologies increase the performance of personnel, require technical maintenance/repair and need professional and conscious use. Moreover, the lowest score was detected in the ‘benefit subscale’. The benefit subscale includes statements regarding the content of health technologies, their areas of use and the benefits they provide in this context. These results showed that the nurses in the sample group had a good awareness of health technologies, they adopted health technologies and they needed to be supported with additional comprehensive information and practices.

This study showed a moderate and significant correlation between the variables of HPHTAAS and NPSES and revealed that HPHTAAS had a significant positive effect on NPSES. Upon the literature review, no study has been found on this subject; however, in their study, P et al. (2024) reported that the combined application of medicine and technology can improve global and local capacities and build a certain level of self‐confidence (P et al. 2024). However, the fact that the study was conducted with intensive care nurses is thought to possibly have affected the outcome since one study reported that nurses who worked in internal medicine clinics were severely anxious about the use of AI, while nurses who worked in intensive care unit were moderately anxious about its use (Nasreldin Othman et al. 2021). On the other hand, another study reported that hospital information technology systems were important in the quality and improvement of nursing service delivery, as well as the effectiveness of nursing staff performance (Lulin et al. 2020).

On the other hand, another finding of this study was that the score of those who worked on mixed shifts (day and night) on the ‘awareness subscale’ of the HPHTAAS was higher than the score of those who worked on permanent night shifts. Considering these differences, rotating night shift nurses to day shifts and reviewing their attitudes towards health technologies may help nurses become more equal and better prepared for increasing technology and AI. Working both day and night in the clinic may have led to a more general evaluation of technologies in the health field. However, it has been reported that permanent night shifts may have a negative effect on the performance of nurses (Di Muzio et al. 2019). It is thought that there is a need for further studies in this field.

### 4.1 Limitations

The most significant limitation of the present study is that the data were collected from the nurses who were working in a single city and a single hospital. Therefore, it is not possible to generalise the results of the study to nurses who work in different hospitals, cities and countries. The assessment tools used have been validated for reliability. However, the results are based on nurses’ self‐reports. To our knowledge, this is the first study to examine the attitudes of intensive care nurses towards health technologies and their relationship with their professional self‐efficacy. This creates limitations in the discussion of the results. On the other hand, another limitation is that data were collected only from nurses who were working in ICUs.

## 5 Conclusions and Implications for Nursing

Intensive care nurses were found to exhibit good attitudes towards health technologies and have a high professional self‐efficacy. Attitude towards health technologies can directly affect professional self‐efficacy positively. It is thought that the findings of this study may guide the prediction of future attitudes of nurses in other groups who are not yet very familiar with health technologies. Since nurses play an important role in the health sector, it is important to know their attitudes towards health technologies. It is recommended to support positive attitudes towards AI and encourage the effective use of AI technologies in nursing practice. By creating an environment that supports innovation and creativity in healthcare services, nurses should be encouraged to develop a proactive attitude towards AI and technological developments. Also, the evaluation of the effect of rapidly advancing health technologies on nurses’ self‐efficacy may provide preliminary information about their adaptation to technology and the efficiency of health technology. It is recommended that similar studies be conducted on different and larger sample groups.

## Author Contributions

Study design: AG. Data collection: KAK. Data analysis: AG. Study supervision: AG. Manuscript writing: AG and KAK. Critical revisions for important intellectual content: AG and KAK.

## Conflicts of Interest

No conflict of interest has been declared by the authors.
